# Growth hormone receptor antagonism downregulates ATP-binding cassette transporters contributing to improved drug efficacy against melanoma and hepatocarcinoma *in vivo*

**DOI:** 10.3389/fonc.2022.936145

**Published:** 2022-07-05

**Authors:** Reetobrata Basu, Yanrong Qian, Samuel Mathes, Joseph Terry, Nathan Arnett, Trent Riddell, Austin Stevens, Kevin Funk, Stephen Bell, Zac Bokal, Courtney Batten, Cole Smith, Isaac Mendez-Gibson, Silvana Duran-Ortiz, Grace Lach, Patricia Alexandra Mora-Criollo, Prateek Kulkarni, Emily Davis, Elizabeth Teaford, Darlene E. Berryman, Edward O. List, Sebastian Neggers, John J. Kopchick

**Affiliations:** ^1^ Edison Biotechnology Institute, Ohio University, Athens, OH, United States; ^2^ Department of Biological Sciences, Ohio University, Athens, OH, United States; ^3^ Russ College of Engineering, Ohio University, Athens, OH, United States; ^4^ Molecular Cellular Biology Program, Ohio University, Athens, OH, United States; ^5^ Department of Biomedical Sciences, Heritage College of Osteopathic Medicine, Ohio University, Athens, OH, United States; ^6^ Translational Biological Sciences Program, Ohio University, Athens, OH, United States

**Keywords:** growth hormone (GH), GHA, growth hormone receptor antagonist (GHRA), melanoma, HCC, ABC transporters, drug resistance

## Abstract

Knockdown of GH receptor (GHR) in melanoma cells *in vitro* downregulates ATP-binding cassette-containing (ABC) transporters and sensitizes them to anti-cancer drug treatments. Here we aimed to determine whether a GHR antagonist (GHRA) could control cancer growth by sensitizing tumors to therapy through downregulation of ABC transporters *in vivo*. We intradermally inoculated Fluc-B16-F10 mouse melanoma cells into GHA mice, transgenic for a GHR antagonist (GHRA), and observed a marked reduction in tumor size, mass and tumoral GH signaling. Moreover, constitutive GHRA production in the transgenic mice significantly improved the response to cisplatin treatment by suppressing expression of multiple ABC transporters and sensitizing the tumors to the drug. We confirmed that presence of a GHRA and not a mere absence of GH is essential for this chemo-sensitizing effect using Fluc-B16-F10 allografts in GH knockout (GHKO) mice, where tumor growth was reduced relative to that in GH-sufficient controls but did not sensitize the tumor to cisplatin. We extended our investigation to hepatocellular carcinoma (HCC) using human HCC cells *in vitro* and a syngeneic mouse model of HCC with Hepa1-6 allografts in GHA mice. Gene expression analyses and drug-efflux assays confirm that blocking GH significantly suppresses the levels of ABC transporters and improves the efficacy of sorafenib towards almost complete tumor clearance. Human patient data for melanoma and HCC show that GHR RNA levels correlate with ABC transporter expression. Collectively, our results validate *in vivo* that combination of a GHRA with currently available anti-cancer therapies can be effective in attacking cancer drug resistance.

## Introduction

The pleiotropic antagonism of growth hormone (GH) and its major downstream effector, insulin-like growth factor 1 (IGF1), in driving proliferative and invasive growth of multiple different types of cancer, especially at the paracrine/autocrine level and with increasing age, has now established it as a serious anti-cancer strategy (1–3). At the epidemiological level, meta-analysis of 23 studies suggests that multiple cancer incidence and mortality is increased in patients with acromegaly (excess GH due to hypersecreting pituitary adenoma) (4). Also, long-term follow-up studies with the Israeli and Ecuadorian cohorts of individuals with Laron Syndrome (LS) (GH resistant due to dysfunctional mutations in GHR) remarkably find them to be completely resistant to all cancers (5, 6). Moreover, numerous studies using GH transgenic (hGH or bGH) mice, GHR antagonist (GHA) transgenic mice, congenital and adult-onset GHR knockout (GHRKO, 6mGHRKO) mice and several mouse models of GH deficiency (Ames, Snell, lit/lit) confirm the tumor driving role of GH and IGF1 and also reveal several IGF1-independent actions of GH in favoring a therapy-resistant and metastatic cancer phenotype (7–12). A series of recent work by us and others has mechanistically described the repertoire of tumor-supportive effects of GH, beyond its well-known growth promoting action.

Melmed and colleagues have elegantly described a critical role of peripheral/non-pituitary GH in the aging colon in abetting a tumor supportive microenvironment (13). An increasing mutational burden due to age or external mutagens in normal cells trigger p53 production, and GH was found to be a p53 target (14–16). In turn, GH suppresses p53 production by a negative feedback loop, suppresses DNA damage repair (DDR) pathways (16, 17), as well as causes an extensive remodeling of the extracellular matrix (ECM) in direct support of tumor progression (13). Additionally, Lobie and colleagues have described in much detail, the anti-apoptotic and cancer stem cell-inducing actions of autocrine GH in breast, liver, endometrial and colorectal cancers (18–27). We have elucidated GH’s role in directly promoting multidrug resistance by upregulation of the ABC multidrug transporter expression in melanoma *in vitro* and *in vivo* (28, 29). Combined work has also corroborated that GH is a critical inducer of the metastatic process of epithelial-to-mesenchymal transition (EMT) in several different cancers (30, 31). In addition to these direct effects of GH on the tumor biology, two indirect but major effects of GH lead further towards detrimental prognoses: (i) hepatic production of >75% of the circulating, potent mitogen and anti-cancer target IGF1, which also promotes a therapy resistant and invasive cancer phenotype (32), and (ii) increasing insulin resistance by exerting a diabetogenic effect, known since the 1930s (33).

ATP binding cassette-containing (ABC) multidrug transporters or multidrug efflux pumps are upregulated in all types of cancers, mediating drug efflux and resistance (34, 35). Previously, we had identified the highest GHR expression in human melanoma cells in the NCI-60 cell lines (36) and also demonstrated that GH upregulates resistance to doxorubicin, cisplatin, paclitaxel, and vemurafenib by increasing the expression of ABCB, ABCC, and ABCG groups of ABC transporters (28). Knockdown of GHR in these melanoma cell lines drastically sensitized these cells to the aforementioned chemo- and targeted therapies (28). Moreover, by inoculating syngeneic B16-F10 tumors in either bGH mice (high serum GH and IGF1) or GHRKO mice (high serum GH, low serum IGF1), we confirmed that GH directly increases ABCB and ABCG type and IGF1 preferentially increases ABCC type of ABC transporters in melanoma (37). Subsequent studies confirmed a GH-regulated ABCG2 dependent docetaxel resistance in human breast cancer xenografts in Nude mice (38). Further, we confirmed autocrine GH expression in B16-F10 tumors grown in C57BL/6J mice (37). Therefore, melanoma presented as an ideal cancer type to verify the hypothesis of whether a GHR antagonist can sensitize the effect of chemotherapy in a suitable mouse model. Here, we used the GHA mice, transgenic for bovine GHR antagonist, for implantation of Fluc-B16-F10 cells and compared their response to cisplatin against the same in wild-type (WT) mice. We also performed an orthogonal validation using cisplatin treatment against B16-F10 implants in the GHKO mice, which lack GH expression. In both cases, decreased or lack of GH action significantly improved treatment efficacy and tumor clearance. Subsequently, we extended our investigation to hepatocellular carcinoma (HCC), which has a poor survival rate and remarkably high drug resistance. Using a syngeneic mouse model of HCC with Hepa1-6 implants and sorafenib treatment in GHA vs. WT mice, we confirmed that GHR antagonism can significantly enhance therapeutic success in cancers expressing the GHR.

## Materials and methods

### Cell culture and reagents

Fluc-B16-F10 mouse melanoma cells (CL052, Imanis Life Sciences, Rochester, MN, USA) and Hepa1-6 mouse hepatocarcinoma (CRL-1830, ATCC, Manassas, VA, USA) were purchased. The cells were maintained in high glucose DMEM, with 10% FBS (complete growth media) and 1× penicillin-streptomycin, in a humidified 5% CO2 incubator at 37°C. Cells were passaged twice a week (passage numbers are <20). Recombinant bovine GH (#CYT-636, ProspecBio, East Brunswick, NJ, USA) and a mouse mGHA synthesized in our laboratory was used *in vitro*. For short-term GH signaling studies, Fluc-B16-F10 cells were seeded on 6-well plates and incubated overnight in complete growth media. On the second day, the media was replaced with serum-free media for 4 hours prior to the treatment with bGH as indicated. In longer-term treatment (48 hours or one week), cells were incubated with bGH in 2% FBS complete growth media (replaced every other day), as previously described (29).

Cisplatin (S1166, Selleckchem.com, Houston, TX, USA) was purchased and prepared in saline for *in vivo* studies. The drug was heated in a water bath at 55°C for 30 min to increase the solubility. The solutions were stored at 4°C for less than one week. Sorafenib was dissolved in dimethyl sulfoxide (DMSO) to produce a 10 mM stock solution. The drug was stored at −20°C for *in vitro* studies. For the *in vivo* study, the solution was prepared immediately before use. To prepare the solvent, 1:1 (v/v) ratio of Cremophor EL (S6828, Selleckchem.com, Houston, TX, USA) and ethanol were mixed first, then the mixture was diluted in water (1:4 v/v). D-luciferin (#88292, D-luciferin monosodium salt, Thermo Fisher Scientific, Waltham, MA, USA) was stored at -20°C and prepared freshly before each IVIS imaging.

### Animal studies

6-month-old male GHA and GHKO mice, as well as their respective WT controls, all in a C57BL/6J genetic background were used (39–41). These syngeneic mice are widely used for the evaluation of Fluc-B16-F10 melanoma or Hepa1-6 HCC *in vivo*. The flanks of mice were trimmed one week before commencement of the experiments. In all melanoma studies, 100,000 Fluc-B16-F10 cells/100uL (1:1 v/v mixture of PBS and Growth Factor Reduced Matrigel Matrix, Corning, #356231, NY, USA) were intradermally inoculated into the flank of each mouse. GHA and GHKO mice were housed with their WT littermate controls. The lengths of perpendicular tumor diameters were measured each day or every other day using a digital caliper as previously described (29). Tumor volume was calculated using the formula: tumor size = 0.5 × (length) × (breadth)^2^.

For the melanoma/GHA/cisplatin study, 6-month-old male GHA and WT mice were used. The mice were assigned into 4 groups: i) WT control (n=8); ii) WT + cisplatin (n=8); iii) GHA alone (n=8); and iv) GHA + cisplatin (combination) (n=8). Similarly, for the combination study between GHKO and cisplatin in melanoma models, we assigned 4 groups: i) WT control (n=6); ii) WT + cisplatin alone (n=6); iii) GHKO mice (n=6); iv) GHKO + cisplatin (n=6). In the melanoma combination studies, cisplatin (5mg/kg body weight) was i.p. injected starting day 8 or 10 every third day for about 2 weeks. Saline was used as solvent.

For the HCC study, 5 million Hepa1-6 cells/100ul (1:1 mixture of PBS and Growth Factor Reduced Matrigel, Corning, #356231, NY, USA) were inoculated subcutaneously in the flanks of 6-month-old female GHA and WT mice. The mice were randomly assigned into 4 groups: i) WT mice (n=6); ii) WT+sorafenib (n=6); iii) GHA alone (n=6); iv) GHA+sorafenib (combination) (n=6). Sorafenib (30mg/kg/day) was delivered by oral gavage. Solvent was 12.5% Cremophor EL/12.5% ethanol/75% water.

From the breeding for the GHA mice we got almost equal number of males and female litters. As we used male mice for the melanoma study, we used the female mice for HCC study, keeping in mind that melanoma has a markedly higher incidence in the male population, while HCC is equally common in males and females (https://gco.iarc.fr/).

Following sacrifice, tumors were surgically removed and frozen in liquid nitrogen and stored at −80°C until RNA and protein were extracted and analyzed. Some of the tumor tissues were fixed in 10% formalin overnight and transferred to 70% ethanol before being processed for IHC. Animal studies were performed in accordance to policies of the Ohio University Institutional Animal Care and Use Committee and fully complied with all federal, state, and local policies.

### *In vivo* imaging system

On the day of IVIS imaging, a fresh stock solution of D-luciferin (#88292, D-luciferin monosodium salt, Thermo Fisher Scientific, Waltham, MA, USA) was prepared at 15 mg/ml in warm PBS. D-luciferin was measured and dissolved in warm PBS and then filtered through a 0.2-μm filtered syringe. Each mouse was weighed on the same day. The injection amount for each mouse was 10 μL/g of body weight (equal to 150mg D-luciferin/kg body weight). The solution was injected i.p. 3 minutes before anesthesia to allow the D-luciferin to be distributed and metabolized in the body. Then, mice were exposed to 2.5% isoflurane in the 2% O_2_ chamber. After that, the mice were transferred to the IVIS chamber under anesthesia. 10 mins after i.p. injection, the total photons from the entirety of the animals’ bodies were counted by using the IVIS imaging system (IVIS 100, Xenogen, PerkinElmer, Waltham, MA, USA) according to the manufacturer’s instructions. The exposure setting was 1 sec. Data were then analyzed using Living Image 3.50 software (Xenogen, PerkinElmer, Waltham, MA, USA). A successful intradermal injection was indicated by images from day zero that show local bioluminescence near the point of inoculation on the flank of each mouse. Images were taken on Day 0 (inoculation), and Days 8,14, and 21 after inoculation.

### Cell viability assays

Approximately ten thousand Fluc-B16-F10 or Hepa1-6 cells were seeded on 96-well plates and incubated overnight in complete growth media. On the second day, the media was replaced with serum-free media for 4 hours. Then, cells were incubated for 48 hours in 2% FBS media with various doses of bGH. After treatment, cell proliferation was determined using a Resazurin Cell Viability Assay (Sigma Aldrich), as described previously (29). For the EC50 assays, Hepa1-6 cells were seeded in 6-well plates and pre-treated with bGH for one week. The media were freshly prepared and replaced every other day. After one week, the cells were gently trypsinized, counted and reseeded on 96-well plates. Then the cells were treated with complete growth media with various doses of sorafenib for 48 hours. After the incubation, cell proliferation was determined.

### *Ex vivo* studies (sera treatment)

For the ex vivo studies, the sera collected from GHA or WT mice were mixed with serum-free DMEM at a ratio of 1:1 (v/v) immediately before addition to cultured cells. For the ex vivo cell viability assay, approximately ten thousand Fluc-B16-F10 cells were seeded on 96-well plates overnight. On the second day, the cells were incubated with mouse sera-DMEM mixture for 24 hours before determination of viability. For real-time RT-qPCR, Fluc-B16-F10 cells were seeded on 6-well plates and incubated with mouse sera-DMEM mixture for 48 hours. Then, cells were washed with warm PBS 3 times before RNA isolation.

### Drug efflux assay

To assess drug efflux rate in human HCC cells, the Vybrant multidrug resistance assay kit (Molecular Probes #V13180, Eugene, OR) was used. The assay uses non-fluorescent calcein acetoxymethylester (calcein-AM) as a drug-mimic and a substrate for cancer cell efflux pumps. Calcein-AM is highly lipid soluble and permeates the cell membrane where it is converted to a fluorescent calcein by the intracellular esterases. The amount of intensely fluorescent calcein that is retained, can be measured as a measure of dye effluxed or an indication of dye retention inside the cell. The assay was performed as per the manufacturer’s protocol with some necessary optimizations. Briefly, the GH treated cells were counted and seeded at 50,000 cells/well in a black, clear bottom Costar 96-well plate (Corning #3603, Corning, NY) and then calcein-AM was added at a final concentration of 2 uM, and incubated at 37C for 2 hr. After thorough washing, fluorescence was measured at 494 (excS)/517 nm (emi) in a spectramax M2 fluorescence plate reader (Molecular Devices, Sunnyvale, CA) and SoftMax Pro v6.2.1 software. Experiments were done in quadruplicate.

### Real-time RT-qPCR

Total RNA from cells and tumor tissues were isolated (GeneJET RNA Purification kit, #K0732 Thermo Fisher Scientific, Waltham, MA, USA), quantified using the BioAnalyzer 2100 (Agilent, Santa Clara, CA, USA), and reverse transcribed to cDNA (Thermo Fisher Scientific Maxima Enzyme Mix, #K1642, 5× Reaction Mix, #R1362) as previously described (29). Power SYBR Green PCR Master Mix (#4367659, Thermo Fisher Scientific, Waltham, MA, USA) was employed to quantitatively measure the abundances of target RNA in the samples using Applied Biosystems (Thermo Fisher Scientific, Waltham, MA, USA). Relative RNA levels are presented in 2^(-ddCt) format and normalized against reference genes (*B2m, Eif3f* or *Hprt*). Fold changes are shown relative to controls.

### Western blotting

Tumor tissues from mice were separately thawed and diluted in 2X RIPA buffer (Sigma, St. Louis, MO) and 2X protease inhibitor cocktail (Sigma, St. Louis, MO). Protein solubilization was achieved through mechanical homogenization using a Precelly’s homogenizer, followed by brief sonication, and centrifugation at 12,000 *g* for 15 min at room temperature. The supernatant protein solutions were transferred to clean tubes. Protein concentrations were measured using Bio-Rad Protein Assay. Based on the amount of protein obtained and the initial weight of each sample, equal amounts of protein was loaded in each lane for western-blot analyses.

To determine GH induced and activated (phosphorylated) intracellular signaling molecules, protein extracts from Fluc-B16-F10 and Hepa1-6 cells and tumors were assayed for STAT5, AKT, ERK1/2, SRC; ABCB1, ABCG2, ABCC1, ABCC2, and ABCC4 (#9351, #4058, #4370, #2101, #94205, #4685, #9102, #2109, #13342, #42078, #72202, #12559, #12857, CST, Danvers, MA, USA); ABCB8 and ABCC9 (#PA5-76139, # PA5-42398, Thermo Fisher Scientific, Waltham, MA, USA); and ABCG1 (NB400-132, Novus Biologicals, Littleton, CO, USA) as previously described (29). β-actin (#4970, CST, Danvers, MA, USA) was used as a loading control. Anti-rabbit IgG, HRP-linked secondary antibody (#7074, CST, Danvers, MA, USA) and SuperSignal West Femto Maximum Sensitivity Substrate (#34095, Thermo Fisher Scientific, Waltham, MA, USA) were used. Densitometry analysis of individual blots was performed using Image Studio LITE Ver 5.2 (LI-COR, Lincoln, NE, USA).

### Enzyme-linked immunosorbent assay

Following blood collection from each mouse, serum IGF1 levels were determined by leaving the blood at room temperature for 30 mins, followed by centrifugation at 8,000xg for 15 mins at 4°C. The sera were then collected and measured by ELISA for IGF1 (#22-IG1MS-E01, ALPCO, Salem, NH, USA) according to the manufacture’s guidelines.

### Bioinformatic analyses of TCGA datasets

Spearman’s correlation analysis of GHR and IGF1 in 371 HCC patients in the TCGA dataset was performed using the LinkedOmics platform (42). The LinkedOmics database contains clinical and multi-omics data and a total of 11,158 patients for 32 cancer types from TCGA project. The survival probabilities of HCC patients with high (above median) and low below median) expression of either IGF1R (cut-off value = 106), or ABCB1 (cut-off value = 1881) or ABCC1 (cut-off value = 342) were evaluated using the KMplotter platform (43). The gene expression correlation analysis and corresponding heatmap generation for 371 HCC patients and 477 melanoma patients from the TCGA dataset was performed using cBioportal platform (44).

### Statistics

All *in vitro* experiments were repeated at least thrice. Student t tests were performed in most experiments. For *in vitro* experiments, data represent mean ± standard deviation. For *in vivo* experiments, data represent mean ± standard errors. Tumor sizes were analyzed by repeated measures (SPSS Statistics 17.0, Chicago, IL, USA). Significance was set as a p value ≤ 0.05.

## Results

### GHRA suppresses mouse melanoma growth *in vivo*

**Figure 1:**
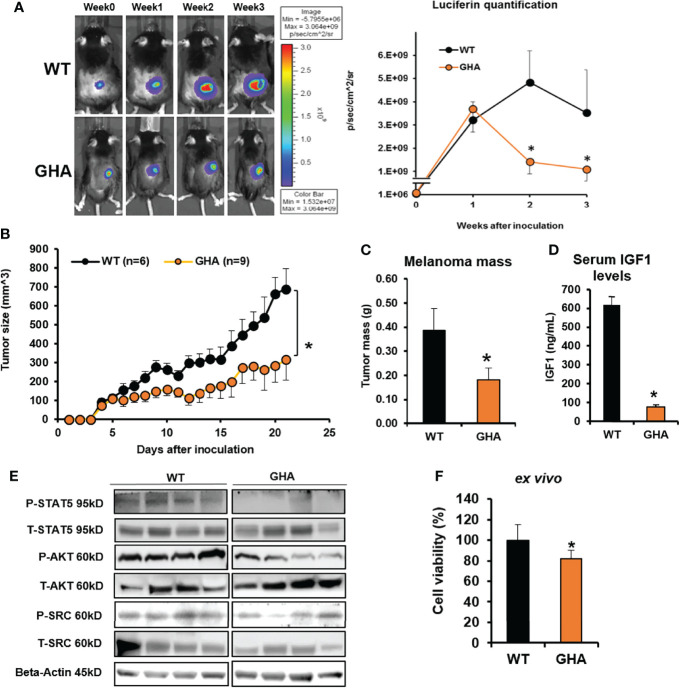
GHRA suppresses syngeneic mouse melanoma growth *in vivo*. **(A)** Mouse Fluc-B16-F10 cells grafted intradermally on the right flank of syngeneic C57BL6/J wild-type (WT) and GHA male mice (transgenic for bGH G119K GHR antagonist) (n=6). Tumor growth over 4-weeks was followed by luminescent imaging following luciferin injections. Luciferin signal was quantified and plotted on the right. The changes in tumor volume (Fluc-B16-F10 in WT and GHA mice) from digital caliper measurement **(B)** and tumor mass **(C)** corroborate the suppressed tumor growth in GHA mice which has markedly lower serum IGF1 levels **(D)** due to presence of a circulating GHRA. Western-blot analysis of the GH downstream signaling mediators – phosphorylated STAT5, AKT and SRC kinase in the tumors of GHA and WT mice **(E)**. B16-F10 cells in culture when treated for 72-hours with serum collected from WT and GHA mice showed suppressed growth rate in the GHA mouse serum **(F)**. (*p < 0.05, mouse studies – repeated measure using SPSS; cell viability - Students t test, n = 3).

Based on our series of *in vitro* studies with human and mouse melanoma cells and *in vivo* study with syngeneic melanoma mouse models of GH excess, here, we hypothesized that a GHR antagonist (GHRA) might successfully suppress melanoma tumor growth *in vivo*. We first confirmed that mouse GHRA (G119K variant of mGH) does suppress bGH induced STAT5 phosphorylation in Fluc-B16-F10 cells (**Supplementary Figure 1**). We proceeded next to perform intradermal inoculation of the same Fluc-B16-F10 cells in a compatible (C57BL/6J background) mouse transgenic for bovine GHRA, the GHA mouse (39, 40). These GHA mice, with elevated GH and suppressed IGF1 in the serum, presented a significant >50% downregulation in the growth of the tumor inoculum over 3-weeks compared to the same in WT mice, as shown by Luciferin levels (**Figure 1A**) and caliper-based assessment of tumor volumes (**Figure 1B**). Moreover, post-dissection tumor masses were also reduced by >50% in the GHA mice compared to the WT (**Figure 1C**). The serum IGF1 levels of the tumor bearing GHA mice were suppressed by 88% compared to that in the WT (**Figure 1D**), confirming the attenuation of GH action at a systemic level due to the endogenous GHRA expression. Suppressed STAT5, AKT and SRC phosphorylation in the GHA tumors compared to the WT tumors further corroborate the effects of transgenic GHRA expression in lowering GH induced signaling pathways in the tumors (**Figure 1E**). Additionally, *in vitro* cell viability of Fluc-B16-F10 cells was 19% lower than when grown in GHA mouse serum compared to WT mouse serum (**Figure 1F**). Collectively the results confirm that a GHRA can successfully attenuate melanoma allograft growth in an *in vivo* setting.

### GHRA markedly sensitizes melanoma tumors to cisplatin treatment *via* downregulation of ABC transporters *in vivo*

**Figure 2:**
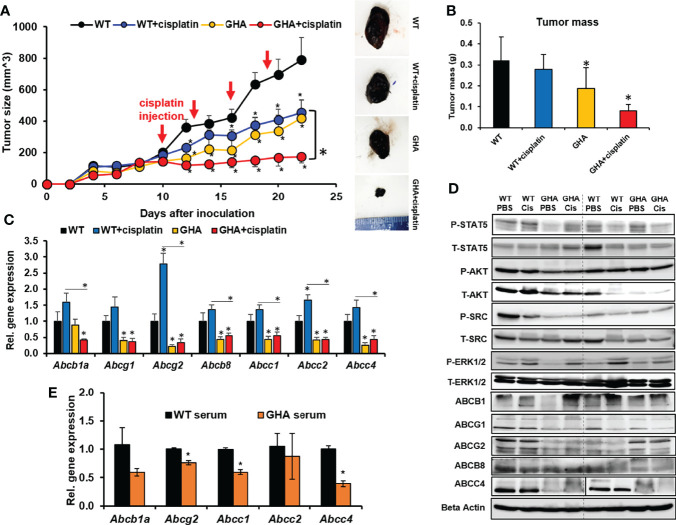
Effect of GHRA on response of syngeneic mouse melanoma tumors to cisplatin treatment *in vivo*. **(A)** Mouse Fluc-B16-F10 cells grafted intradermally on the right flank of syngeneic C57BL6/J wild-type (WT) and GHA mice (transgenic for bGH G119K GHR antagonist) (n=8). The changes in tumor volume (Fluc-B16-F10 in WT and GHA mice) from digital caliper measurement and representative tumors post-dissection **(A)** and tumor mass **(B)** corroborate suppressed tumor growth in GHA mice and improved tumoral response to cisplatin in the GHA mice. The qPCR analysis of ABC transporter RNA expression involved in multi-drug efflux from the tumors in WT and GHA mice **(C)** and western-blot assessment of GH downstream signaling and ABC transporter protein levels **(D)** are shown. **(E)** The changes in ABC transporter RNA level in B16-F10 cells in culture when treated with serum collected from WT and GHA mice (*p < 0.05, mouse studies – repeated measure using SPSS; other assays - Students t test, n = 3).

To corroborate whether GHR antagonism can sensitize melanoma to chemotherapy, as indicated by our earlier *in vitro* studies, we intradermally inoculated GHA and WT mice with Fluc-B16-F10 cells on the flank. The allografted WT and GHA mice were further divided into two groups each – one treated with saline and the other treated with cisplatin following tumor stabilization (day 10 post-inoculum) for 15 days. Cisplatin inhibited the tumor size in the WT mice by 44% while tumor growth in the GHA mice was suppressed by 48% of the WT mice (**Figure 2A**). Remarkably, cisplatin treatment achieved a pronounced reduction in tumor volume in the GHA mice, and the tumor volumes in GHA mice were only 22% of the same in cisplatin treated WT mice (**Figure 2A**). Dissected tumor weights further confirm the lowest tumor mass in the cisplatin treated GHA mice (**Figure 2B**). There are a number of mechanisms by which the tumors in GHA mice could have been sensitized to the cisplatin treatment, of which we specifically looked at GH induced changes in ABC-multidrug transporter pump expression, which was found to be a principal mode of GH regulated chemoresistance from our earlier *in vitro* studies (28, 29). We found that the RNA expression of *Abcb1a, Abcg1, Abcg2, Abcb8, Abcc1, Abcc2, and Abcc4* multidrug transporters in the GHA mouse tumors were markedly decreased compared with the same in WT mice for both saline and cisplatin treatment groups (**Figure 2C**). Cisplatin treatment specifically increased the *Abcb1a, Abcg2*, and *Abcc2* transporter levels in the WT mice, which was not observed in case of cisplatin treatment in the GHA mice (**Figure 2B**). Western blot confirmed the suppressed GH signaling, including phosphorylated STAT5, AKT, SRC, and ERK1/2, in tumors from the GHA mouse groups (**Figure 2D**). Also, protein levels of multidrug efflux transporters ABCG1, ABCG2, and ABCC4 in tumors from GHA mouse groups were decreased compared to that found in the WT groups for both saline and cisplatin treatments (**Figure 2D**). Ex vivo studies treating cultured Fluc B16-F10 cells with sera from WT or GHA mice lead to downregulation of *Abcb1a*, *Abcg2*, *Abcc1* and *Abcc4* (**Figure 2E**). Collectively, the results confirm that a GHRA can significantly sensitize melanoma allografts to chemotherapy treatments in an *in vivo* setting, *via* a marked downregulation of ABC multidrug efflux transporter expression.

### Lack of endogenous GH suppresses melanoma allografts but does not sensitize melanoma tumors to cisplatin treatment

**Figure 3:**
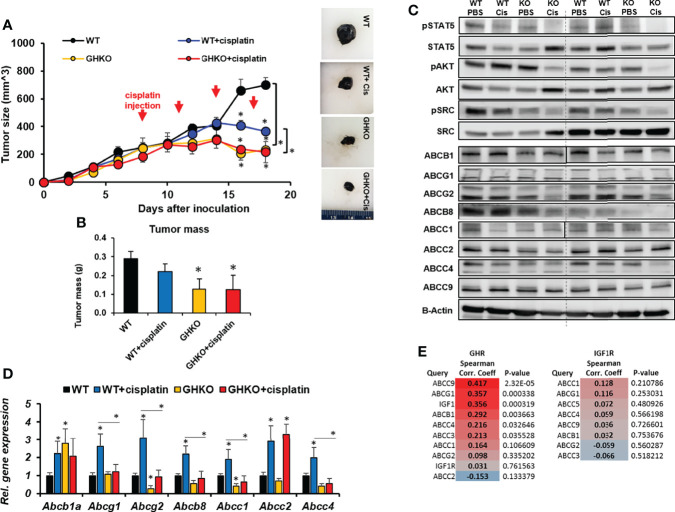
Effect of absence of GH on response of syngeneic mouse melanoma tumors to cisplatin treatment *in vivo*. **(A)** Mouse Fluc-B16-F10 cells were grafted intradermally on the right flank of syngeneic C57BL6/J wild-type (WT) and GH knockout (GHKO) mice (n=6). The changes in tumor volume (Fluc-B16-F10 in WT and GHKO mice) from digital caliper measurement and representative tumors post-dissection **(A)** and tumor mass **(B)** corroborate suppressed tumor growth in GHKO mice but not improved tumoral response to cisplatin in the GHKO mice. Western-blot assessment of GH downstream signaling and ABC transporter protein levels **(C)** and qPCR analysis of ABC transporter RNA expression involved in multi-drug efflux from the tumors in WT and GHKO mice **(D)** are shown. (*p < 0.05, mouse studies – repeated measure using SPSS; other assays - Students t test, n = 3). **(E)** Spearman correlation analysis for transcript levels of GHR and ABC transporter and IGF1R and ABC transporters in 471 human melanoma patients in the TCGA cohort (generated using Linkedomics).

As an orthogonal confirmation of our observation in the GHA mice, we investigated if a congenital absence of GH action can similarly affect chemotherapeutic efficacy in melanoma using GH knockout (GHKO) mice. GHKO mice have a congenital absence of GH in all tissues and consequently, very low levels of IGF1 (41). We inoculated Fluc-B16-F10 cells intradermally in the flanks of WT or GHKO mice, each of which were again split into two groups and treated with either cisplatin or saline. Cisplatin alone inhibited the tumor size in WT mice by about 50% of that of untreated controls, whereas cisplatin efficacy was higher in GHKO mice where the tumor sizes were only 31% of that of untreated controls and significantly (59%) lower than that of cisplatin treated WT mice (**Figure 3A**). The trend was identical in the post-dissection tumor weights also, where the GHKO tumors were significantly smaller than those in WT mice, irrespective of cisplatin treatment (**Figure 3B**). Interestingly, although cisplatin treatment efficacy increased in the presence of GHRA compared to GHRA alone in the GHA mice (**Figure 2A**), here, cisplatin treatment in GHKO mice was not more efficacious than in untreated GHKO animals (**Figure 3A**). This indicates the following possibility: chemoresistance induced by tumor-derived GH is effectively suppressed by endogenous GHRA in GHA mice thereby improving cisplatin efficacy in those animals, while the absence of GHRA in GHKO mice, did not suppress tumor-derived GH effects and did not improve cisplatin efficacy. Therefore, suppressing autocrine GH is essential to improve chemotherapeutic efficacy which can be achieved by a direct antagonism of GHR.

Similar to the observations in GHA vs WT mice, the phosphorylation states of GH-induced signaling mediators STAT5, SRC, and AKT were also markedly reduced in the tumors from GHKO mice compared to the same from WT mice (**Figure 3C**). RNA levels of *Abcg2, Abcb8, Abcc1, Abcc2, and Abcc4* were suppressed in the GHKO mice (**Figure 3D**) while protein levels of ABCB1, ABCG1, ABCG2, ABCB8, ABCC1, ABCC2, and ABCC4 were significantly lowered in GHKO mice compared to the WT mice (**Figure 3C**). A query of human tumor transcriptomic data for 471 melanoma patients from The Cancer Genome Atlas (TCGA) dataset revealed a positive correlation of *IGF1* and *ABCC9, ABCG1, ABCB1*, and *ABCC4* with *GHR* expression, and *ABCC1* with both *GHR* and *IGF1R* expression (**Figure 3E**), supporting our observations.

### Targeting GHR significantly improves response of human hepatocellular carcinoma cells to anti-cancer drugs *in vitro*

**Figure 4:**
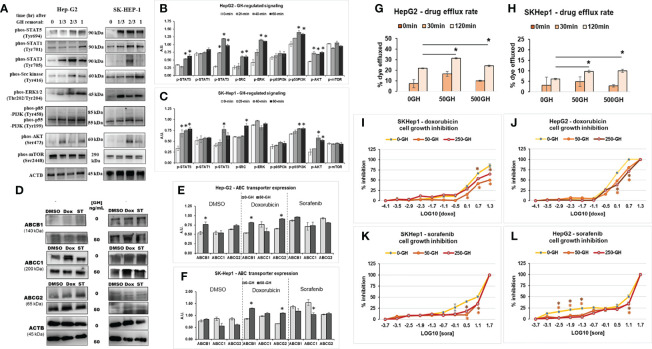
GH signaling drives drug resistance in human HCC cells. **(A)** Treatment with exogenous GH (50ng/mL) causes activation of STATs 3 and 5, SRC family kinase, ERK1/2 and PI3K-AKT signaling in human HCC cells - Hep-G2 **(B)** and SK-Hep-1 **(C)** cells in culture. **(D)** Doxorubicin or sorafenib tosylate (ST) and/or GH treatment increases ABCB1, ABCC1 and ABCG2 protein expression in human HCC cells – Hep-G2 **(E)** and SK-Hep-1 **(F)** in culture. **(G, H)** Recombinant human GH treatment (at 50 or 500ng/mL) increases drug efflux rate in human HCC cells. **(I–L)** Recombinant hGH (at 50 or 250ng/mL) suppresses doxorubicin induced growth inhibition in SK-Hep-1 **(I)** and Hep-G2 cells **(J)** and sorafenib induced growth inhibition in SK-Hep1 **(K)** and Hep-G2 cells **(L)**. (*p < 0.05, Students t test, n = 3).

In context of the improved response to chemotherapy in combination with attenuated GH action in our earlier *in vitro* and current *in vivo* studies in melanoma, we further hypothesized that other cancer types with GHR expression may also be amenable to this therapy sensitizing effects. We focused on liver cancer (hepatocellular carcinoma; HCC), which has a high lethality, compounded by intense therapy resistance and very few available effective therapeutic approaches (45). Additionally, prior *in vitro* and *in vivo* reports confirm an active GH/IGF axis in HCC. First, we conducted an *in vitro* study using human liver cancer cell lines SK-Hep-1 and HepG2. Treatment with recombinant hGH induced robust phosphorylation for STAT5, STAT3, SRC, ERK1/2, p55-PI3K, and AKT within 20-minutes to 1 hour of GH treatment (**Figures 4A–C**). We also found that protein levels of ABCB1, ABCC1 and ABCG2 are increased following doxorubicin and sorafenib treatment in the human HCC cells which was emphasized by GH treatment (**Figures 4D–F**). Moreover, GH treatment caused a significant increase in drug efflux rate in both the cell lines (**Figures 4G, H**). We next assessed if cell growth inhibition by the anti-cancer drugs is inhibited by GH induced increase in drug efflux rate. We indeed observed that cell growth inhibition by doxorubicin or sorafenib was markedly suppressed by increasing doses of GH treatment (**Figures 4I–L**). In fact, treatment with FDA-approved GHR antagonist, Pegvisomant, effected a 2-fold reduction in the EC50 of doxorubicin against human HCC cells irrespective of exogenous GH treatment (**Supplementary Figure 3**). interestingly, exogenous hGH treatment did not increase doxorubicin EC50, but pegvisomant treatment reduced the same drastically. This could be due to pegvisomant mediated antagonism of autocrine GH action. By qPCR, we detected GH1 gene transcripts in both HepG2 and SK-Hep-1 cells (not shown here). These results validate our hypothesis that GHR inhibition in GHR expressing HCC cells can be effective in improving response to therapy. To evaluate the success of these *in vitro* results in an *in vivo* setting, we next employed the GHA mouse model.

### GHRA markedly sensitizes HCC tumors to sorafenib treatment *via* downregulation of ABC transporters *in vivo*

**Figure 5:**
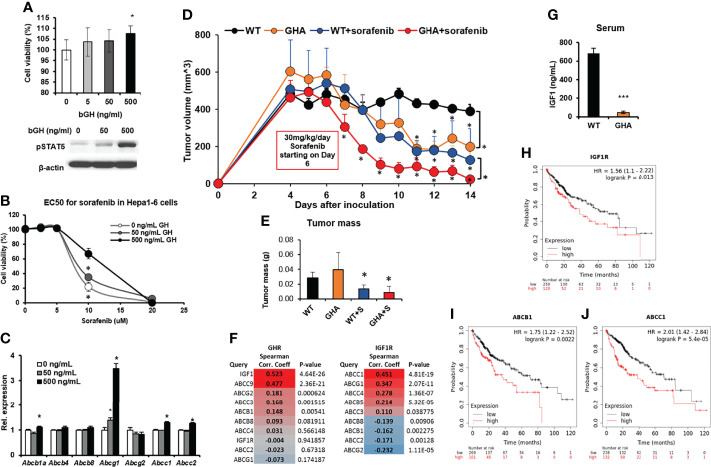
Effect of GHRA on response of syngeneic mouse hepatocellular carcinoma tumors to sorafenib treatment in vivo. **(A)** Mouse Hepa1-6 hepatocellular carcinoma (HCC) cells respond to bovine GH stimulation in culture showing increased cell viability and STAT5 activation after 72 hours **(A)** as well as increase in the EC50 dose of sorafenib **(B)**. Additionally, GH treatment increased ABC transporter levels in the cultured Hepa1-6 cells **(C)**. Mouse Hepa1-6 cells grafted subcutaneously on the right flank of syngeneic C57BL6/J wild-type (WT) and GHA mice (transgenic for bGH G119K GHR antagonist) (n=6). The changes in tumor volume (Hepa1-6 in WT and GHA mice) from digital caliper measurement **(D)** and post-dissection tumor mass **(E)** show suppressed tumor growth and improved tumoral response to sorafenib in the GHA mice. **(F)** Spearman correlation analysis for transcript levels of GHR and ABC transporter and IGF1R and ABC transporters in the tumor of 371 HCC patients in the TCGA cohort. **(G)** Serum IGF1 levels of Hepa1-6 tumor-bearing GHA mice are significantly lower than that of tumor-bearing WT mice. **(H–J)** Correlation of overall survival probability of 371 HCC patients in the TCGA cohort with RNA expression of IGF1R **(H)**, or ABCB1 **(I)**, or ABCC1 **(J)**. (*p < 0.05, ***p < 0.001, mouse studies – repeated measure using SPSS; other assays - Students t test, n = 3).

Mouse hepatocarcinoma cells Hepa1-6 express GHR, and bGH treatment for 48 hours increases cell viability in a dose-dependent manner, with 500ng/mL bGH showing a significant increase (**Figure 5A**). Moreover, bGH treatment increases sorafenib EC50, also in a dose-dependent manner, in the Hepa1-6 cells in culture from 7.5uM to 12.5 uM (with 500ng bGH) (**Figure 5B**). RNA expression analysis shows marked upregulation of *Abcb1a, Abcg1, Abcc1*, and *Abcc2* in presence compared to absence of bGH, during sorafenib treatment (**Figure 5C**). We made subcutaneous inoculation of Hepa1-6 cells in the flanks of GHA and WT mice in two groups and treated with either saline or sorafenib following establishment of actively growing tumors. Sorafenib inhibited the tumor size in the WT mice by 75% while tumor growth in the untreated GHA mice was suppressed by 51% of the untreated WT mice (**Figure 5D**). Sorafenib treatment also achieved a pronounced reduction in tumor volume in the GHA mice, where the tumor volumes were only 19% of that of sorafenib treated WT mice and 6% of that of untreated WT mice (**Figure 5D**). Dissected tumor weights further confirm the lowest tumor mass in the sorafenib treated GHA mice (**Figure 5E**). By the end of the study, the tumors in the combination group were quite small and, in some cases, undetectable. Further, the RNA expression of *Abcb1a, Abcg1, Abcg2, Abcc1, and Abcc4* in HCC tumors from GHA or combination group were significantly decreased compared with WT group (**Supplementary Figure 2**).

It is important to note that normal liver expresses a very high level of GHR (and very little or no IGF1R), and GH induced hepatic production of IGF1 accounts for almost 70-80% of circulating IGF1 (46). In HCC, the liver tumors overexpress IGF1R, which is now directly fueled by the GH-induced hepatic IGF1 in the tumor milieu. Therefore, in HCC, GH and IGF1 actions jointly create a highly proliferative and therapy resistant tumor phenotype. Correlation (Spearman) analysis of human HCC patients in the TCGA dataset revealed that *GHR* and *IGF1* mRNA levels were strongly and significantly correlated (R = 0.523, p = 4.64e-26) (**Figure 5F**). Importantly, serum IGF1 levels were significantly suppressed due to GHRA, in the tumor bearing mice (**Figure 5G**). In human HCC patients, *IGF1R* expression was negatively correlated with patient survival (Hazard ratio = 1.56, p = 0.013), wherein patients with high *IGF1R* levels survived significantly less than patients with low-*IGF1R* levels (38 vs. 71 months) (**Figure 5H**). Human tumor transcriptomic data for 377 HCC patients also show a positive correlation of multiple ABC transporters including the *ABCB1, ABCC3, ABCC9* and *ABCG2* with GHR expression, while *ABCC1,ABCC4 and ABCG1* levels correlated with *IGF1R* expression (**Figure 5H**), confirming our *in vivo* observations. Further, the GH-regulated *ABCB1* and IGF1-regulated *ABCC1* are significantly and negatively correlated with HCC patient survival (ABCB1: 36 vs. 70 months, Hazard ratio = 1.75, p = 0.0022; ABCC1: 38 vs. 71 months, Hazard ratio = 2.01, p = 5.4e-05) (**Figures 5I, J**).

## Discussion

Resistance to therapy remains a major hurdle in cancer treatment (47). Recent research established that GH regulates multiple key mechanisms of making tumors resistant to anti-cancer drugs (chemotherapy and targeted therapy) including but not limited to increasing tumoral drug efflux *via* ABC transporters, inducing epithelial-to-mesenchymal transition (EMT), suppressing apoptosis, increasing cancer stem cells (CSCs), and inducing fibrosis and extracellular matrix (ECM) remodeling (31). Several mouse models, as well as Pegvisomant treatment in Nude mouse xenografts, have confirmed that GHR antagonism is an effective monotherapy in slowing tumor growth (3). Despite significant heterogeneity in patient treatment types, the TCGA melanoma patient dataset confirms a positive correlation of *GHR* and ABC transporters. Yet, tumoral response to GHR antagonism coupled with any anti-cancer chemo- or targeted therapy in an *in vivo* setting had not been studied. Therefore, in light of our series of *in vitro* observations in melanoma, we first hypothesized that targeting GHR in melanoma can improve the response of the tumor to anti-cancer drugs by attenuating ABC transporter expression in a pre-clinical mouse model of melanoma. Here, we validated our hypothesis by employing the GHA mice, transgenic for a murine GHRA (G119K bGH), vs. the WT mice. In addition to a marked suppression of melanoma implants compared to that in the WT mice, the presence of GHRA in the GHA mice significantly increased the tumoral sensitivity to cisplatin treatment. The GHRA-cisplatin combination in the GHA mice maximally limited tumor mass and volume and showed consistent suppression of multiple ABC-transporter expression levels, known to mediate cisplatin efflux (48). Our extended study in melanoma with the GHKO mice allowed us to critically distinguish the effect of absence of GH vs presence of GHRA in sensitizing melanoma to cisplatin effect. As our orthogonal experiments revealed, cisplatin efficacy did not further reduce the suppressed tumor growth in the GHKO mice. This can be attributed to existing autocrine GH from the tumor, which could still promote tumoral ABC transporter expression and consequent cisplatin efflux in the GHKO mice but was successfully attenuated in the GHA mice by the circulating GHRA. This is exemplified by ABCC2, a cisplatin transporter, which was suppressed in cisplatin treated GHA mice, but remained upregulated in the cisplatin-treated GHKO mice. These results collectively indicate that a systemic presence of GHRA can be transformative in improving anti-cancer therapeutic efficacy in melanoma.

GH action has been repeatedly implicated in hepatic malignancies, which have very low 5-year survival rate, highly limited therapeutic options, and a high degree of therapy resistance. Hepatocellular carcinoma (HCC) expresses higher levels of GHR than normal liver hepatocytes (49). In a cohort of HCC patients, higher human *GH* mRNA expression in HCC tumors is associated with overall worse survival (20). Autocrine human GH promotes ABCG2 expression and confers properties of CSCs in human HCC cells in culture (18). Mice transgenic for either human or bovine GH (hGH or bGH mice respectively), show increased hepatic hypertrophy, hyperplasia, and pro-tumorigenic signaling leading to an increased incidence of spontaneous liver tumors (50–52). Furthermore, treatment with the hepatocarcinogen diethylnitrosamine induces 2.6-fold and 4-fold higher liver tumor formation in male and female bGH mice, respectively, compared to age-matched controls (53). In light of our promising results in melanoma, we next hypothesized that GHRA can also sensitize HCC cells to anti-cancer therapy. Sorafenib, a tyrosine kinase inhibitor, is a currently approved first-line targeted therapy for advanced HCC (54). Sorafenib inhibits multiple receptor tyrosine kinases including c-RAF, wild type and mutant B-RAF, c-KIT, vascular endothelial growth factor receptors (VEGFR1, VEGFR2, VEGFR3, platelet-derived growth factor receptor ß (PDGFR ß) and FMS-like tyrosine kinase 3 (FLT-3) (55). Several of these kinases hyperactivate tumor cell signaling and angiogenesis, while suppressing apoptosis. Yet, sorafenib treatment of HCC patients has a low response rate or only prolongs patient’s life by months due to drug resistance (54, 56). Previous research shows that ABCB1 and ABCG2 mediates sorafenib resistance in HCC (57, 58). In our study here, we validate that circulating GHRA inhibits the gene expression of ABCB1 and ABCG2 in the HCC tumors and markedly sensitizes tumors to sorafenib effect to the extent of complete remission in some of the experimental mice. Wang et al. also show that inhibition of IGF1R enhances the efficacy of sorafenib in inhibiting the growth and proliferation HCC cells (59); although the alteration of ABC transporters was not reported. Moreover, IGF1 signaling is known to upregulate ABCB1, ABCG2, ABCC1, and ABCC2 multidrug efflux pumps (60) and can thus elicit an indirect effect of GH in promoting drug efflux. We had recently shown that ABCB and ABCG type efflux pumps to be more directly regulated by GH while ABCC group of efflux pumps are directly regulated by IGF1, using multiple mouse models of GH/IGF1 axis (29). Indeed, GH exerts several IGF-independent direct actions as well as IGF-dependent indirect actions on the tumor cell, inducing therapy resistance, metastases and invasive proliferation and relapse (31). Therefore, GHRA exerts a dual effect of inhibiting not only the direct binding of GH to the tumoral GHR, but also disconnects the GH-induced IGF1 supply to the tumoral IGF1R. This is particularly important for HCC for two reasons: (i) there is a massive expression of IGF1R following hepatic malignancy while normal liver tissues do not express IGF1R (46), and (ii) normal liver tissue overexpresses GHR and GH induced hepatic IGF1 production accounts for as much as 75-80% of circulating IGF1 (61) – also a paracrine fuel for the tumoral IGF1R. Therefore, the pronounced improvement of sorafenib efficacy in GHA mice, as well as marked suppression of HCC allografts even in untreated GHA mice can be reasonably hypothesized to be a cumulative effect of GHR inhibition and lower IGF1 levels.

The association of GH action with chemotherapeutic failure in cancer is longstanding. We have previously shown that siRNA-mediated GHR targeting in cultured melanoma cells markedly sensitize them to cisplatin, doxorubicin, paclitaxel or vemurafenib effects by attenuating ABCB, ABCG, and ABCC type multidrug transporters and provided a new mechanistic rationale of GH-mediated therapy refractoriness (28). We also showed that GH-induced increase in ABC transporter expression promotes melanosomal drug sequestration (37) and primes B16-F10 allografts for chemoresistance in bGH or GHRKO mice by upregulating multiple ABC transporters, even in the absence of drug treatments (29). Subsequent studies in Nude mice reported that GH-directed ABCG2 expression promoted docetaxel resistance in breast cancer (38). In breast and endometrial cancers, autocrine GH had earlier been shown to blunt the efficacy of several chemotherapy treatments (mitomycin-C, doxorubicin, cisplatin, arsenic trioxide, ruxolitinib) wherein the effects were ascribed to a suppressed apoptosis (23, 62–64). More recently, chemically-induced mammary tumors established in wild-type rats and GH supplemented spontaneous dwarf rats responded to doxorubicin treatment with regression of tumors in the dwarf animals when GH supplementation was stopped, whereas poor doxorubicin response was observed in GH-sufficient wild-type animals (65). Together with these exemplary reports, our current study provides robust *in vivo* proof of concept that tumoral sensitization to anti-cancer drugs is achievable by the presence of a circulating GHRA, as in the GHA mice.

It is well known that GHRA suppresses GH action and lowers serum IGF1 levels in a dose-dependent manner (66, 67). The GHA mouse, transgenic for a GHRA and with reduced IGF1, mimics a pharmacologic inhibition of GHR and provides an excellent model to assess the effects of multiple chemotherapeutic efficacies in presence of a GHRA for multiple cancer types. However, the studies are limited to a murine tumor cell line and C57BL6 compatibility, but allows for evaluating therapeutic prognosis in a syngeneic, immunocompetent model. In the GHA mice, the serum GHRA levels are about 2 ug/mL (68). Clinically, serum levels of Pegvisomant in treated individuals with acromegaly are about 2.2-5.6 ug/mL (69). Therefore, the serum levels of GHRA in Pegvisomant-treated human patients and in the GHA mouse are comparable. We note that the constitutive expression of the GHRA in this mouse line can have a pre-treatment effect which compounds estimation of a possible required dose for GHRA in cancer patients. This is an obvious limitation of this model and should be considered prior to translation of the results to human patients. Pegvisomant is a specific antagonist of the human GHR and harbors nine different amino acid changes, which, along with 5-6 PEG moieties, reduces its binding affinity to the mouse GHR, necessitating quite high doses to reduce hepatic IGF1 levels and use in xenograft studies (70–72). In such cases, the GHA mouse can be a useful *in vivo* platform to assess therapeutic efficacy of combining GHRA with anti-cancer therapies. Although previous studies have used the GHA vs. WT mice to compare chemically-induced mammary and liver cancer developments (3), in view of the recent understanding of GH in regulating therapy resistance, this mouse model appears to be a valuable tool for future investigations. Additional studies, beyond our work, aimed towards more directly establishing a causal role of GH action in inducing ABC transporter expression in chemoresistant cells might assess increases of GH regulated transcription factor bindings at upstream of ABC transporter genes as well as confirming the GH action by selective ablations of ABC transporter expression in cells.

In conclusion, our study provides a first pre-clinical validation of the hypothesis that targeting GHR can improve anti-cancer therapeutic success *in vivo* for two different human cancers associated with marked drug resistance and high lethality – melanoma and hepatocellular carcinoma. We show that a circulating GHRA successfully and significantly improves cisplatin (in melanoma) or sorafenib (in HCC) effects and acts as a springboard for future investigations directed at assessing treatments with approved GHR antagonists and anti-cancer drugs for GHR-expressing human cancers. The translation of these results to the clinic is feasible, given the availability of FDA-approved GHR antagonist Pegvisomant, which is currently approved for acromegaly and known to successfully normalize serum IGF1 in >90% of the patients (73). The promising results of combining GHRA with chemotherapy observed here, not only indicates an improved tumor clearance, but also an opportunity to investigate the lowering of chemotherapy dosages and minimizing the associated side-effects in thousands of cancer patients with GH-responsive tumors.

## Data availability statement

The original contributions presented in the study are included in the article/**Supplementary Material**. Further inquiries can be directed to the corresponding author.

## Ethics statement

The animal study was reviewed and approved by Institutional Animal Care and Use Committee, Ohio University.

## Author contributions

Conceptualization: YQ, RB, SN, JJK; Data curation: YQ, RB, SD, SM, JJK; Formal Analysis: YQ, RB, SM, SD; Funding acquisition: YQ, JT, NA, JJK; Investigation: YQ, RB, SM, JT, NA, TR, AS, ZB, CB, CS, IM, PM, SD, GL, PK, ET, ED, JJK; Methodology: YQ, RB, NA, ZB, CS, SD, ED, ET; Project administration: YQ, JJK; Resources: KF, SB, EL, DB, JJK; Software: YQ, RB, ZB, SD, ED; Supervision: JJK; Validation: YQ, RB, SM, SD, JJK; Visualization: YQ, RB, SM, NA, ZB, GL; Writing – original draft: RB, YQ; Writing – review & editing: RB, YQ, SM, EL, DB, SN, JJK.

## Funding

This work was supported, in part, by the State of Ohio’s Eminent Scholar Program that includes a gift from Milton and Lawrence Goll to JK, National Institutes of Health (NIH)-R01AG059779 to JK; Ohio University Research Council, Baker Fund, and PACE Funds from Ohio University to YQ; the Provost Undergraduate Research Funds and the John J. Kopchick Molecular Cell Biology/Translational Biomedical Sciences Undergraduate Student Support Funds to NA and JT; Student Enhancement Award and Dr. Sig Maier Undergraduate Research Fund to JT; the AMVETS, the Edison Biotechnology and Diabetes Institutes at Ohio University.

## Conflict of interest

The authors declare that the research was conducted in the absence of any commercial or financial relationships that could be construed as a potential conflict of interest.

## Publisher’s note

All claims expressed in this article are solely those of the authors and do not necessarily represent those of their affiliated organizations, or those of the publisher, the editors and the reviewers. Any product that may be evaluated in this article, or claim that may be made by its manufacturer, is not guaranteed or endorsed by the publisher.

## References

[1] Perry JK Wu ZS Mertani HC Zhu T Lobie PE. Tumour-derived human growth hormone as a therapeutic target in oncology. Trends Endocrinol Metab (2017) 28:587–96. doi: 10.1016/J.TEM.2017.05.003 28622965

[2] Boguszewski CL Boguszewski MCDS. Growth hormone’s links to cancer. Endocr Rev (2019) 40:558–74. doi: 10.1210/ER.2018-00166 30500870

[3] Basu R Qian Y Kopchick JJ. MECHANISMS IN ENDOCRINOLOGY: Lessons from growth hormone receptor gene-disrupted mice: Are there benefits of endocrine defects? Eur J Endocrinol (2018) 178:R155–81. doi: 10.1530/EJE-18-0018 29459441

[4] Dal J Leisner MZ Hermansen K Farkas DK Bengtsen M Kistorp C. Cancer incidence in patients with acromegaly: A cohort study and meta-analysis of the literature. J Clin Endocrinol Metab (2018) 103:2182–8. doi: 10.1210/JC.2017-02457 29590449

[5] Steuerman R Shevah O Laron Z. Congenital IGF1 deficiency tends to confer protection against post-natal development of malignancies. Eur J Endocrinol (2011) 164:485–9. doi: 10.1530/EJE-10-0859 21292919

[6] Guevara-Aguirre J Balasubramanian P Guevara-Aguirre M Wei M Madia F Cheng CW. Growth hormone receptor deficiency is associated with a major reduction in pro-aging signaling, cancer, and diabetes in humans. Sci Transl Med (2011) 3:70ra13. doi: 10.1126/SCITRANSLMED.3001845 PMC3357623 21325617

[7] Qian Y Berryman DE Basu R List EO Okada S Young JA. Mice with gene alterations in the GH and IGF family. Pituitary (2022) 25:1–51. doi: 10.1007/S11102-021-01191-Y 34797529 PMC8603657

[8] Duran-Ortiz S List EO Ikeno Y Young J Basu R Bell S. Growth hormone receptor gene disruption in mature-adult mice improves male insulin sensitivity and extends female lifespan. Aging Cell (2021) 20:e13506. doi: 10.1111/ACEL.13506 34811874 PMC8672790

[9] Törnell J Carlsson B Pohjanen P Wennbo H Rymo L Isaksson O. High frequency of mammary adenocarcinomas in metallothionein promoter-human growth hormone transgenic mice created from two different strains of mice. J Steroid Biochem Mol Biol (1992) 43:237–42. doi: 10.1016/0960-0760(92)90213-3 1525063

[10] Ikeno Y Hubbard GB Lee S Cortez LA Lew CM Webb CR. Reduced incidence and delayed occurrence of fatal neoplastic diseases in growth hormone receptor/binding protein knockout mice. J Gerontol A Biol Sci Med Sci (2009) 64:522–9. doi: 10.1093/GERONA/GLP017 PMC2667132 19228785

[11] Wang Z Prins GS Coschigano KT Kopchick JJ Green JE Ray VH. Disruption of growth hormone signaling retards early stages of prostate carcinogenesis in the C3(1)/T antigen mouse. Endocrinology (2005) 146:5188–96. doi: 10.1210/EN.2005-0607 16141391

[12] Zhang X Mehta RG Lantvit DD Coschigano KT Kopchick JJ Green JE. Inhibition of estrogen-independent mammary carcinogenesis by disruption of growth hormone signaling. Carcinogenesis (2007) 28:143–50. doi: 10.1093/carcin/bgl138 16916863

[13] Chesnokova V Melmed S. Growth hormone in the tumor microenvironment. Arch Endocrinol Metab (2019) 63:568–75. doi: 10.20945/2359-3997000000186 PMC7025769 31939481

[14] Chesnokova V Zonis S Zhou C Recouvreux MV Ben-Shlomo A Araki T. Growth hormone is permissive for neoplastic colon growth. Proc Natl Acad Sci USA (2016) 113:E3250–9. doi: 10.1073/PNAS.1600561113 PMC4988562 27226307

[15] Chesnokova V Zhou C Ben-Shlomo A Zonis S Tani Y Ren SG. Growth hormone is a cellular senescence target in pituitary and nonpituitary cells. Proc Natl Acad Sci USA (2013) 110:e3331–9. doi: 10.1073/PNAS.1310589110 PMC3761588 23940366

[16] Chesnokova V Zonis S Apostolou A Estrada HQ Knott S Wawrowsky K. Local non-pituitary growth hormone is induced with aging and facilitates epithelial damage. Cell Rep (2021) 37:110068. doi: 10.1016/J.CELREP.2021.110068 PMC8716125 34910915

[17] Chesnokova V Zonis S Barrett R Kameda H Wawrowsky K Ben-Shlomo A. Excess growth hormone suppresses DNA damage repair in epithelial cells. JCI Insight (2019) 4:e125672. doi: 10.1172/JCI.INSIGHT.125762 PMC6413789 30728323

[18] Chen YJ You ML Chong QY Pandey V Zhuang QS Liu DX. Autocrine human growth hormone promotes invasive and cancer stem cell-like behavior of hepatocellular carcinoma cells by stat3 dependent inhibition of claudin-1 expression. Int J Mol Sci (2017) 18:1274. doi: 10.3390/IJMS18061274 PMC5486096 28617312

[19] Chen YJ Zhang X Wu ZS Wang JJ Lau AYC Zhu T. Autocrine human growth hormone stimulates the tumor initiating capacity and metastasis of estrogen receptor-negative mammary carcinoma cells. Cancer Lett (2015) 365:182–9. doi: 10.1016/J.CANLET.2015.05.031 26070963

[20] Kong X Wu W Yuan Y Pandey V Wu Z Lu X. Human growth hormone and human prolactin function as autocrine/paracrine promoters of progression of hepatocellular carcinoma. Oncotarget (2016) 7:29465–79. doi: 10.18632/ONCOTARGET.8781 PMC5045410 27102295

[21] Zhang W Qian P Zhang X Zhang M Wang H Wu M. Autocrine/paracrine human growth hormone-stimulated microrna 96-182-183 cluster promotes epithelial-mesenchymal transition and invasion in breast cancer. J Biol Chem (2015) 290:13812–29. doi: 10.1074/JBC.M115.653261 PMC4447958 25873390

[22] Bougen NM Steiner M Pertziger M Banerjee A Brunet-Dunand SE Zhu T. Autocrine human GH promotes radioresistance in mammary and endometrial carcinoma cells. Endocr Relat Cancer (2012) 19:625–44. doi: 10.1530/ERC-12-0042 22807498

[23] Bougen NM Yang T Chen H Lobie PE Perry JK. Autocrine human growth hormone reduces mammary and endometrial carcinoma cell sensitivity to mitomycin C. Oncol Rep (2011) 26:487–93. doi: 10.3892/OR.2011.1305 21567106

[24] Tang JZ Kong XJ Banerjee A Muniraj N Pandey V Steiner M. STAT3alpha is oncogenic for endometrial carcinoma cells and mediates the oncogenic effects of autocrine human growth hormone. Endocrinology (2010) 151:4133–45. doi: 10.1210/EN.2010-0273 20668024

[25] Brunet-Dunand SE Vouyovitch C Araneda S Pandey V Vidal LJP Print C. Autocrine human growth hormone promotes tumor angiogenesis in mammary carcinoma. Endocrinology (2009) 150:1341–52. doi: 10.1210/EN.2008-0608 18974274

[26] Pandey V Perry JK Mohankumar KM Kong XJ Liu SM Wu ZS. Autocrine human growth hormone stimulates oncogenicity of endometrial carcinoma cells. Endocrinology (2008) 149:3909–19. doi: 10.1210/EN.2008-0286 PMC2488240 18450952

[27] Perry JK Mohankumar KM Emerald BS Mertani HC Lobie PE. The contribution of growth hormone to mammary neoplasia. J Mammary Gland Biol Neoplasia (2008) 13:131–45. doi: 10.1007/S10911-008-9070-Z PMC2665193 18253708

[28] Basu R Baumgaertel N Wu S Kopchick JJ. Growth hormone receptor knockdown sensitizes human melanoma cells to chemotherapy by attenuating expression of ABC drug efflux pumps. Horm Cancer (2017) 8:143–56. doi: 10.1007/S12672-017-0292-7 PMC10355985 28293855

[29] Qian Y Basu R Mathes SC Arnett NA Duran-Ortiz S Funk KR. Growth hormone upregulates mediators of melanoma drug efflux and epithelial-to-mesenchymal transition *in vitro* and *in vivo* . Cancers (Basel) (2020) 12:1–26. doi: 10.3390/CANCERS12123640 PMC7761932 33291663

[30] Brittain AL Basu R Qian Y Kopchick JJ. Growth hormone and the epithelial-to-mesenchymal transition. J Clin Endocrinol Metab (2017) 102:362–3673. doi: 10.1210/JC.2017-01000 28938477

[31] Basu R Kopchick JJ. The effects of growth hormone on therapy resistance in cancer. Cancer Drug Resist (Alhambra Calif) (2019) 2:827–46. doi: 10.20517/CDR.2019.27 PMC7204541 32382711

[32] Ekyalongo RC Yee D. Revisiting the IGF-1R as a breast cancer target. NPJ Precis Oncol (2017) 1:14. doi: 10.1038/S41698-017-0017-Y 29152592 PMC5687252

[33] BA HOUSSAY. The hypophysis and metabolism. N Engl J Med (1936) 214:961–71. doi: 10.1056/nejm193605142142001

[34] Choi Y Yu A-M. ABC Transporters in multidrug resistance and pharmacokinetics, and strategies for drug development. Curr Pharm Des (2014) 20:793–807. doi: 10.2174/138161282005140214165212 23688078 PMC6341993

[35] Chen Z Shi T Zhang L Zhu P Deng M Huang C. Mammalian drug efflux transporters of the ATP binding cassette (ABC) family in multidrug resistance: A review of the past decade. Cancer Lett (2016) 370:153–64. doi: 10.1016/J.CANLET.2015.10.010 26499806

[36] Sustarsic EG Junnila RK Kopchick JJ. Human metastatic melanoma cell lines express high levels of growth hormone receptor and respond to GH treatment. Biochem Biophys Res Commun (2013) 441:144–50. doi: 10.1016/J.BBRC.2013.10.023 PMC3855845 24134847

[37] Basu R Kulkarni P Qian Y Walsh C Arora P Davis E. Growth hormone upregulates melanocyte-inducing transcription factor expression and activity *via* JAK2-STAT5 and SRC signaling in GH receptor-positive human melanoma. Cancers (Basel) (2019) 11:1352. doi: 10.3390/CANCERS11091352 PMC6769493 31547367

[38] Arumugam A Subramani R Nandy SB Terreros D Dwivedi AK Saltzstein E. Silencing growth hormone receptor inhibits estrogen receptor negative breast cancer through ATP-binding cassette sub-family G member 2. Exp Mol Med (2019) 51:1–13. doi: 10.1038/S12276-018-0197-8 PMC6323053 30617282

[39] Chen WY White ME Wagner TE Kopchick JJ. Functional Antagonism Between Endogenous mouse growth hormone (GH) and a GH analog results in dwarf transgenic mice. Endocrinology (1991) 129:1402–8. doi: 10.1210/ENDO-129-3-1402 1874179

[40] Chen WY Wight DC Mehta BV Wagner TE Kopchick JJ. Glycine 119 of bovine growth hormone is critical for growth-promoting activity. Mol Endocrinol (1991) 5:1845–52. doi: 10.1210/MEND-5-12-1845 1791834

[41] List EO Berryman DE Buchman M Jensen EA Funk K Duran-Ortiz S. GH knockout mice have increased subcutaneous adipose tissue with decreased fibrosis and enhanced insulin sensitivity. Endocrinology (2019) 160:1743–56. doi: 10.1210/EN.2019-00167 PMC6760334 31099824

[42] Vasaikar SV Straub P Wang J Zhang B. LinkedOmics: Analyzing multi-omics data within and across 32 cancer types. Nucleic Acids Res (2018) 46:D956–63. doi: 10.1093/NAR/GKX1090 PMC5753188 29136207

[43] Nagy Á Munkácsy G Győrffy B. Pancancer survival analysis of cancer hallmark genes. Sci Rep (2021) 11:6047. doi: 10.1038/S41598-021-84787-5 33723286 PMC7961001

[44] Cerami E Gao J Dogrusoz U Gross BE Sumer SO Aksoy BA. The Cbio cancer genomics portal: An open platform for exploring multidimensional cancer genomics data. Cancer Discovery (2012) 2:401–4. doi: 10.1158/2159-8290.CD-12-0095 PMC3956037 22588877

[45] Anwanwan D Singh SK Singh S Saikam V Singh R. Challenges in liver cancer and possible treatment approaches. Biochim Biophys Acta Rev Cancer (2020) 1873:188314. doi: 10.1016/J.BBCAN.2019.188314 31682895 PMC6981221

[46] Pivonello C De Martino MC Negri M Cuomo G Cariati F Izzo F. The GH-IGF-SST system in hepatocellular carcinoma: Biological and molecular pathogenetic mechanisms and therapeutic targets. Infect Agent Cancer (2014) 9:27. doi: 10.1186/1750-9378-9-27 25225571 PMC4164328

[47] Vasan N Baselga J Hyman DM. A View on drug resistance in cancer. Nature (2019) 575:299–309. doi: 10.1038/s41586-019-1730-1 31723286 PMC8008476

[48] Galluzzi L Senovilla L Vitale I Michels J Martins I Kepp O. Molecular mechanisms of cisplatin resistance. Oncogene (2012) 31:1869–83. doi: 10.1038/ONC.2011.384 21892204

[49] García-Caballero T Mertani HM Lambert A Gallego R Fraga M Pintos E. Increased expression of growth hormone and prolactin receptors in hepatocellular carcinomas. Endocrine (2000) 12:265–71. doi: 10.1385/ENDO:12:3:265 10963047

[50] Snibson KJ. Hepatocellular kinetics and the expression of growth hormone (GH) in the livers and liver tumours of GH-transgenic mice. Tissue Cell (2002) 34:88–97. doi: 10.1016/S0040-8166(02)00012-5 12165243

[51] Snibson KJ Bhathal PS Hardy CL Brandon MR Adams TE. High, persistent hepatocellular proliferation and apoptosis precede hepatocarcinogenesis in growth hormone transgenic mice. Liver (1999) 19:242–52. doi: 10.1111/J.1478-3231.1999.TB00042.X 10395045

[52] Martinez CS Piazza VG González L Fang Y Bartke A Turynl D. Mitogenic signaling pathways in the liver of growth hormone (GH)-overexpressing mice during the growth period. Cell Cycle (2016) 15:748–59. doi: 10.1080/15384101.2016.1148844 PMC4845918 27028000

[53] Snibson KJ Bhathal PS Adams TE. Overexpressed growth hormone (GH) synergistically promotes carcinogen-initiated liver tumour growth by promoting cellular proliferation in emerging hepatocellular neoplasms in female and male GH-transgenic mice. Liver (2001) 21:149–58. doi: 10.1034/J.1600-0676.2001.021002149.X 11318985

[54] Llovet JM Ricci S Mazzaferro V Hilgard P Gane E Blanc J-F. Sorafenib in advanced hepatocellular carcinoma. N Engl J Med (2008) 359:378–90. doi: 10.1056/NEJMOA0708857 18650514

[55] Keating GM Santoro A. Sorafenib: A review of its use in advanced hepatocellular carcinoma. Drugs (2009) 69:223–40. doi: 10.2165/00003495-200969020-00006 19228077

[56] Tang W Chen Z Zhang W Cheng Y Zhang B Wu F. The mechanisms of sorafenib resistance in hepatocellular carcinoma: Theoretical basis and therapeutic aspects. Signal Transduct Target Ther (2020) 5:87. doi: 10.1038/S41392-020-0187-X PMC7292831 32532960

[57] Huang WC Hsieh YL Hung CM Chien PH Chien YF Chen LC. BCRP/ABCG2 inhibition sensitizes hepatocellular carcinoma cells to sorafenib. PloS One (2013) 8:e83627. doi: 10.1371/JOURNAL.PONE.0083627 PMC3877048 24391798

[58] Wang H Qian Z Zhao H Zhang X Che S Zhang H. CSN5 silencing reverses sorafenib resistance of human hepatocellular carcinoma HepG2 cells. Mol Med Rep (2015) 12:3902–8. doi: 10.3892/MMR.2015.3871 26035694

[59] Wang F Bank T Malnassy G Arteaga M Shang N Dalheim A. Inhibition of insulin-like growth factor 1 receptor enhances the efficacy of sorafenib in inhibiting hepatocellular carcinoma cell growth and survival. Hepatol Commun (2018) 2:732–46. doi: 10.1002/HEP4.1181 PMC5983153 29881824

[60] Yuan J Yin Z Tao K Wang G Gao J. Function of insulin-like growth factor 1 receptor in cancer resistance to chemotherapy. Oncol Lett (2018) 15:41–7. doi: 10.3892/OL.2017.7276 PMC5738696 29285186

[61] Al-Samerria S Radovick S. The role of insulin-like growth factor-1 (IGF-1) in the control of neuroendocrine regulation of growth. Cells (2021) 10:2664. doi: 10.3390/CELLS10102664 34685644 PMC8534318

[62] Gentilin E Minoia M Bondanelli M Tagliati F degli Uberti EC Zatelli MC. Growth hormone differentially modulates chemoresistance in human endometrial adenocarcinoma cell lines. Endocrine (2017) 56:621–32. doi: 10.1007/S12020-016-1085-4 27585662

[63] Minoia M Gentilin E Molè D Rossi M Filieri C Tagliati F. Growth hormone receptor blockade inhibits growth hormone-induced chemoresistance by restoring cytotoxic-induced apoptosis in breast cancer cells independently of estrogen receptor expression. J Clin Endocrinol Metab (2012) 97:E907–16. doi: 10.1210/JC.2011-3340 22442272

[64] Zatelli MC Minoia M Molè D Cason V Tagliati F Margutti A. Growth hormone excess promotes breast cancer chemoresistance. J Clin Endocrinol Metab (2009) 94:3931–8. doi: 10.1210/JC.2009-1026 19622619

[65] Lantvit DD Unterberger CJ Lazar M Arneson PD Longhurst CA Swanson SM. Mammary tumors growing in the absence of growth hormone are more sensitive to doxorubicin than wild-type tumors. Endocrinology (2021) 162:bqab013. doi: 10.1210/ENDOCR/BQAB013 33475144 PMC7881836

[66] Kopchick JJ. Lessons learned from studies with the growth hormone receptor. Growth Horm IGF Res (2016) 28:21–5. doi: 10.1016/J.GHIR.2015.06.003 26216709

[67] Kopchick JJ List EO Kelder B Gosney ES Berryman DE. Evaluation of growth hormone (GH) action in mice: Discovery of GH receptor antagonists and clinical indications. Mol Cell Endocrinol (2014) 386:34–45. doi: 10.1016/J.MCE.2013.09.004 24035867 PMC3943600

[68] Asa SL Coschigano KT Bellush L Kopchick JJ Ezzat S. Evidence for Growth Hormone (GH) Autoregulation in pituitary somatotrophs in GH antagonist-transgenic mice and GH receptor-deficient mice. Am J Pathol (2000) 156:1009–15. doi: 10.1016/S0002-9440(10)64968-1 PMC1876832 10702416

[69] Madsen M Fisker S Feldt-Rasmussen U Andreassen M Kristensen LO Ørskov H. Circulating levels of pegvisomant and endogenous growth hormone during prolonged pegvisomant therapy in patients with acromegaly. Clin Endocrinol (Oxf) (2014) 80:92–100. doi: 10.1111/CEN.12239 23650996

[70] Divisova J Kuiatse I Lazard ZW Weiss H Vreeland F Hadsell DL. The growth hormone receptor antagonist pegvisomant blocks both mammary gland development and MCF-7 breast cancer Xenograft growth. Breast Cancer Res Treat (2006) 98:315–27. doi: 10.1007/S10549-006-9168-1 16541323

[71] McCutcheon IE Flyvbjerg A Hill H Li J Bennett WF Scarlett JA. Antitumor activity of the growth hormone receptor antagonist pegvisomant against human meningiomas in nude mice. J Neurosurg (2001) 94:487–92. doi: 10.3171/JNS.2001.94.3.0487 11235955

[72] Dagnaes-Hansen F Duan H Rasmussen LM Friend KE Flyvbjerg A. Growth hormone receptor antagonist administration inhibits growth of human colorectal carcinoma in nude mice. Anticancer Res (2004) 24:3735–42. 15736405

[73] Paisley AN Trainer P Drake W. Pegvisomant: A novel pharmacotherapy for the treatment of acromegaly. Expert Opin Biol Ther (2004) 4:421–5. doi: 10.1517/14712598.4.3.421 15006735

