# Supplementary material for: Growth hormone receptor antagonism downregulates ATP-binding cassette transporters contributing to improved drug efficacy against melanoma and hepatocarcinoma in vivo
Source: Front Oncol. 2022 Jul 5;12:936145. doi: 10.3389/fonc.2022.936145 (PMC9296106; doi:10.3389/fonc.2022.936145)
Supplement: Supplementary file 1 [file DataSheet_1.pdf]

Supplementary Figure 1

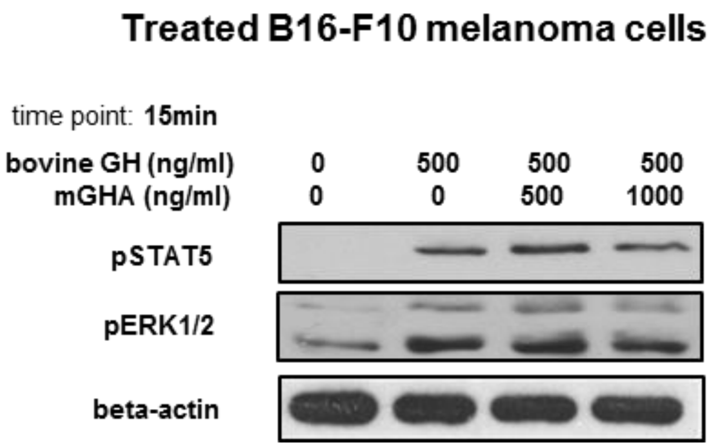

Sup-Fig-1: Mouse melanoma cells B16-F10 were treated with either PBS (control) or bovine GH (500 ng/mL) or both bGH and mouse GHR antagonist (mGHA, @ 500 or 1000 ng/mL) for 15-mins. Following treatment, protein was collected, quantified and western-blot was performed for phosphorylated-STAT5 or phosphorylated-ERK1/2 and beta-Actin.

Supplementary Figure 2

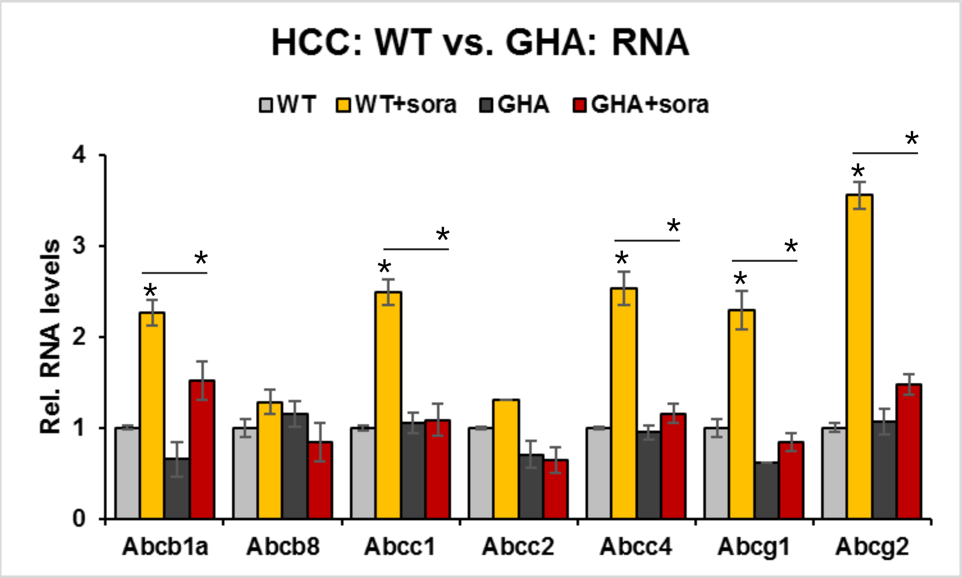

Sup-Fig-2: Mouse HCC cells Hepa1-6 allografts in WT or GHA mice and treated with either PBS or sorafenib, were dissected at the end of study, RNA extracted, and RNA expression of target genes were quantified by RT-qPCR. (*, $p < 0.05$, $n = 3$)

Supplementary Figure 3

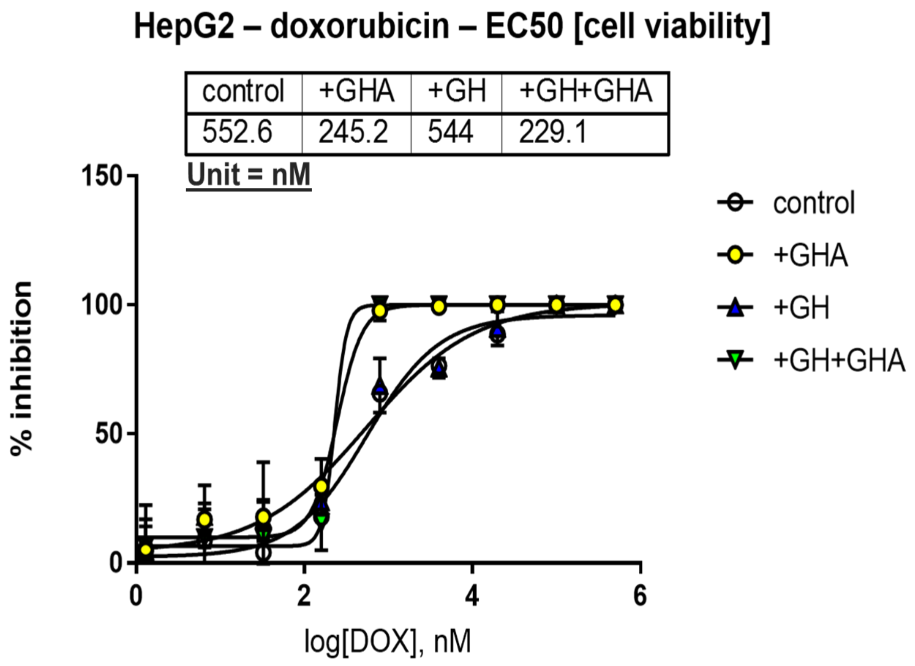

Sup-Fig-3: Human liver cancer cells Hep-G2 were treated with either saline (control) or GH (2.5nM) or GHR antagonist (GHA, @ 500nM) or both GH and GHA along with different doses of chemotherapy doxorubicin for 72-hrs. Cell viability was quantified using Resazurin cell viability assay (as described in Methods section) and EC50 was calculated using GraphPad Prism.

Supplementary Figure 4

Sup-Fig-4: Mouse body-weights across treatments. (A) WT and GHA mice with B16-F10 allografts and treated with saline or cisplatin (Main Fig 2). (B) WT and GHKO mice with B16-F10 allografts and treated with saline or cisplatin (Main Fig 3).

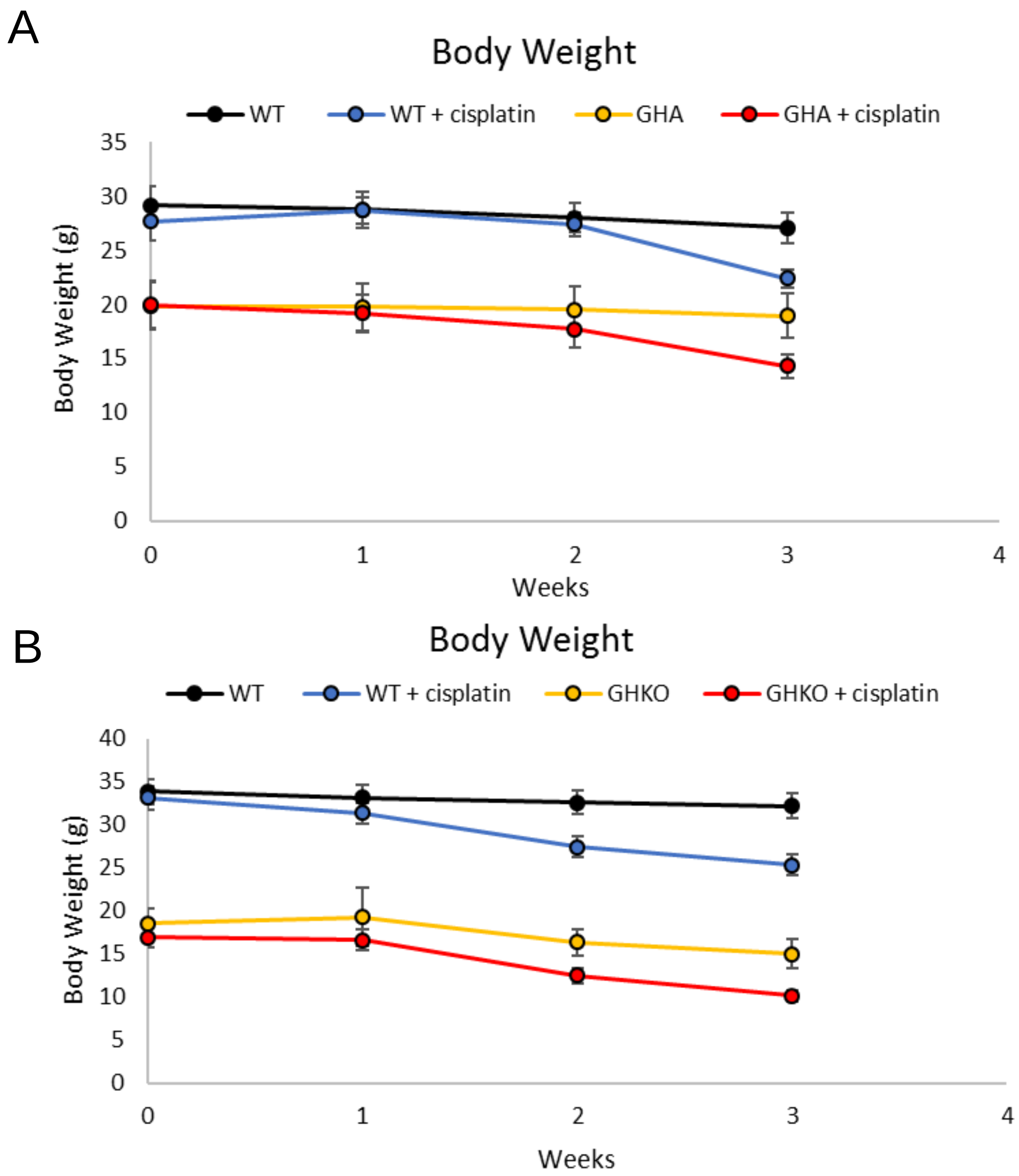

Supplementary Figure 5

Primer sequences for RT-qPCR.

Gene	Full name	Forward(5'->3')	Reverse(5'->3')
Ghr	Mouse growth hormone receptor	GCCTGGGGACAAGTTCTTCTGGA	TGCAGCTTGTCGTTGGCTTTCCC
Igf-1	Mouse insulin like growth factor 1	GACAAACAAGAAAACGAAGC	ATTTGGTAGGTGTTTCGATG
Igf-1r	Mouse insulin like growth factor 1 receptor	AGAACCGAATCATCATAACG	TTTTAAATGGTGCCTCCTTG
Abcb1a	ATP Binding Cassette Subfamily B Member 1A	AGGACAAAAGAAGGAACTTG	GATAAGGAGAAAAGCTGCAC
Abcb8	ATP Binding Cassette Subfamily B Member 8	GCTGTAAAGCAGAAGAACTG	CCAAGACCATACAGTTGAAAG
Abcc1	ATP Binding Cassette Subfamily C Member 1	GTCTATCGTAAGGCTCTTTTG	GACCAGATCATGTTAATGTACG
Abcc2	ATP Binding Cassette Subfamily C Member 2	CGTATATAAGAAGGCACTAACC	CAATCTGTAAAACACTGGACC
Abcc4	ATP Binding Cassette Subfamily C Member 4	AAACAAAGTCATCCTGTTCG	CAGAAAGTTCTTGATCCTCC
Abcg1	ATP Binding Cassette Subfamily G Member 1	CCAGCTTTATGTCCTAAGTC	CACTTCCATGACAAAGTCTG
Abcg2	ATP Binding Cassette Subfamily G Member 2	AAGAGCCAGTCTATGTTACC	AAACTCCAGCTCTATTTTGC
B2m	Beta-2 microglobulin	CTGGTCTTTCTATATCCTGGCT	CATGTCTCGATCCCAGTAGAC
Elf3f	Eukaryotic translation initiation factor 3, subunit F	TACGAACGCCGCAACGAGGG	TGGCACCGAAAAGCAGTTGGTGA
Hprt	Hypoxanthine phosphoribosyltransferase 1	ATCAGTCAACGGGGGACATA	AGAGGTCCTTTTCACCAGCA

Original Western-blot Images

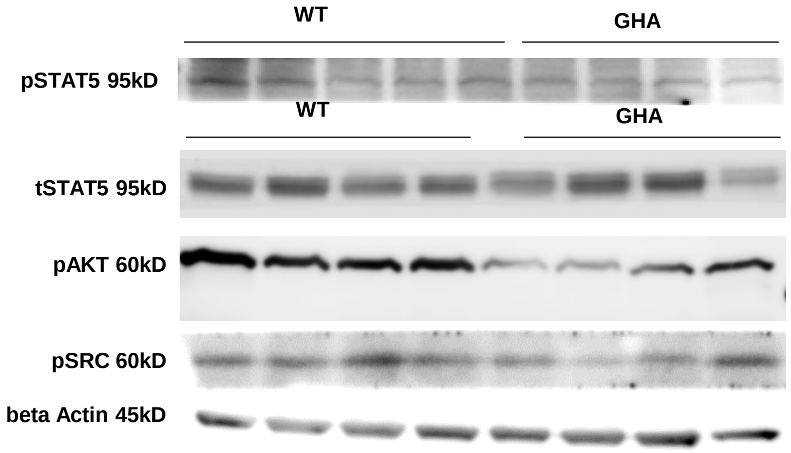

Main Figure 1

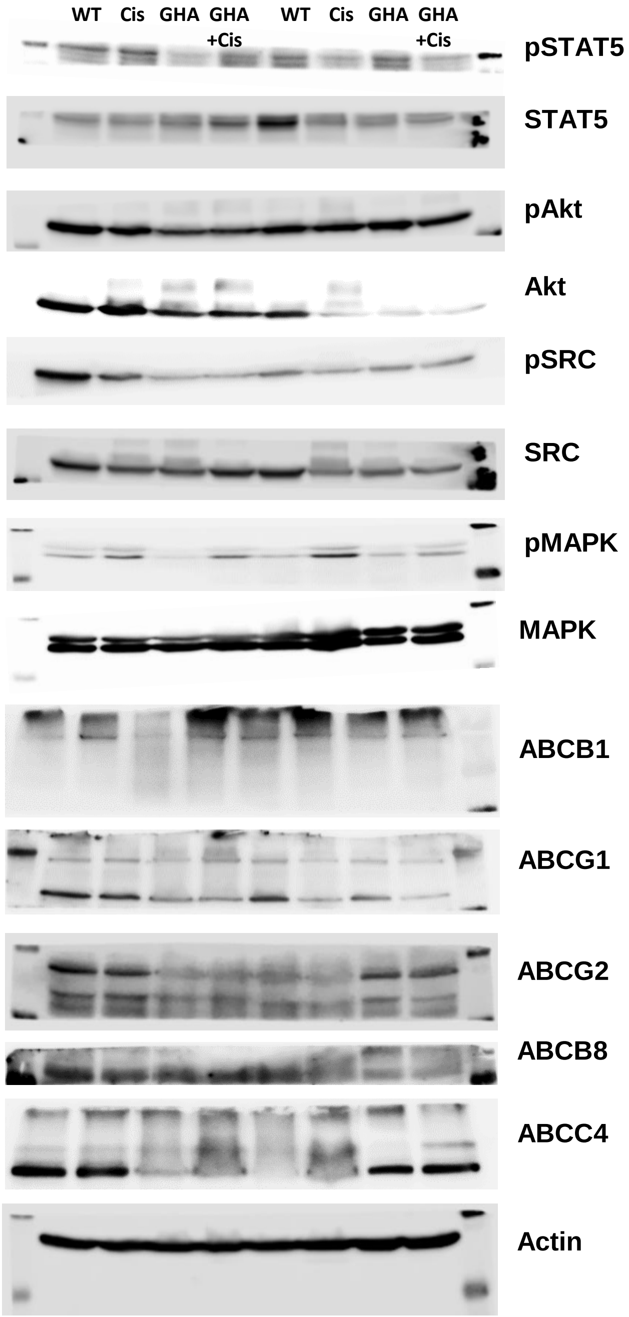

Main Figure 2

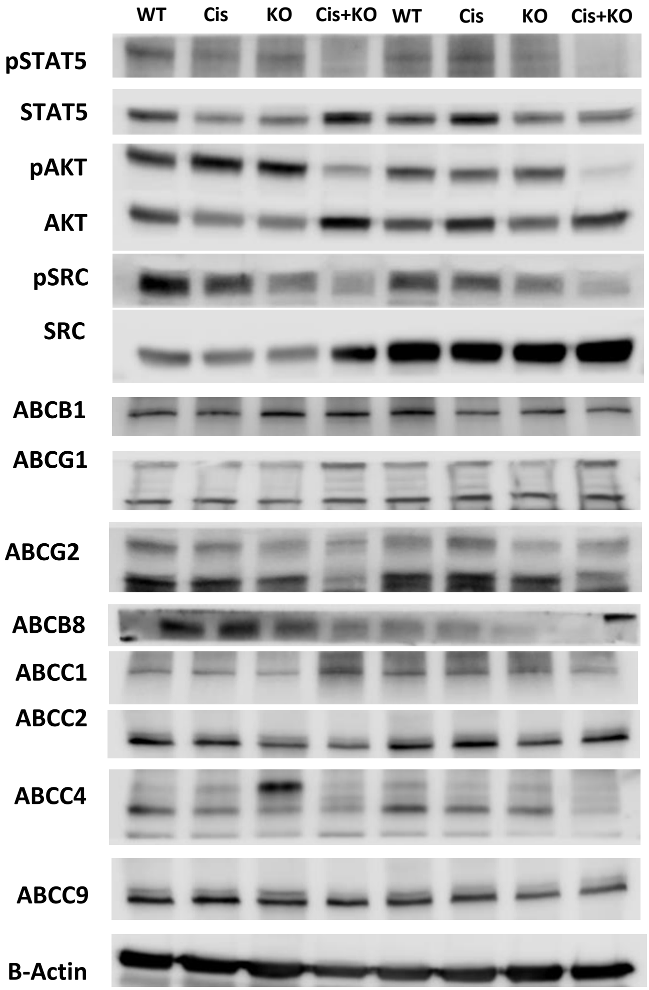

Main Figure 3
